# Long-term reductions in disease impact in patients with chronic migraine following preventive treatment with eptinezumab

**DOI:** 10.1186/s12883-022-02774-3

**Published:** 2022-07-08

**Authors:** Andrew Blumenfeld, Anders Ettrup, Joe Hirman, Bjarke Ebert, Roger Cady

**Affiliations:** 1 The Los Angeles Headache Center, Los Angeles, CA USA; 2 H. Lundbeck A/S, Copenhagen, Denmark; 3 Pacific Northwest Statistical Consulting, Woodinville, WA USA; 4 Lundbeck LLC, Deerfield, IL USA; 5 RK Consults, MO 65721 Ozark, USA; 6 Missouri State University, Springfield, MO USA

**Keywords:** Eptinezumab, Chronic migraine, Patient-reported outcomes, CGRP monoclonal antibody

## Abstract

**Background:**

Eptinezumab is an anti-calcitonin gene-related peptide humanized monoclonal antibody approved for the preventive treatment of migraine in adults. The PREVAIL study demonstrated a favorable safety profile with sustained reductions in overall migraine-related burden in patients with chronic migraine (CM). This post hoc analysis aimed to examine item-level changes in the Migraine Disability Assessment (MIDAS) questionnaire over 2 years in participants with CM on eptinezumab treatment.

**Methods:**

PREVAIL was an open-label, phase 3 trial that included 96 weeks of treatment where 128 adults received intravenous eptinezumab administered over 30 min every 12 weeks (wks) for up to 8 doses of 300 mg. MIDAS was administered at baseline, Wk12, and every 12wks thereafter. Two supplementary MIDAS items not included in the total score calculation assessed number of headache days in the past 3 months (MIDAS headache) and average headache pain severity (from 0 [none] to 10 [worst]). MIDAS total scores were summed from 5 items, each quantifying the number of days in the past 3 months with migraine-related disability. Items 1, 3, and 5 assessed absenteeism, namely how many days the patient missed work/school (Q1), household work (Q3), or family/social/leisure activities (Q5). Items 2 and 4 were measures of presenteeism, namely how many days the patient had reduced productivity in work/school (Q2) or household work (Q4).

**Results:**

Mean MIDAS headache days decreased from 47.4 (baseline) to 17.1 (Wk12) and 16.3 (Wk104). The average headache pain severity score (0‒10) decreased from a mean of 7.3 (baseline) to 5.5 (Wk12) to 4.5 (Wk104). Mean MIDAS scores measuring absenteeism (Q1, 3, 5) changed from 9.7 days at baseline to 3.2 days (Wk12) and to 3.9 days (Wk104). Mean MIDAS scores measuring presenteeism (Q2, 4) at Wk12 decreased from 14.2 days at baseline to 5.2 days (Wk12, 104). Patients categorized with very severe MIDAS disability had a mean total MIDAS score of 84.8, with an average reduction of 56.7 days (Wk12), which was maintained at 32 days at Wk104.

**Conclusions:**

Long-term treatment with eptinezumab in patients with CM suggested sustained reductions in MIDAS-quantified disability, consistent with the sustained reductions in headache frequency and pain severity.

**Trial registration:**

ClinicalTrials.gov identifier: NCT02985398.

**Supplementary Information:**

The online version contains supplementary material available at 10.1186/s12883-022-02774-3.

## Introduction

Chronic migraine (CM) is a disease that results in long-term disability and high disease burden. Migraine is one of the top five leading causes of disability among people aged 10‒49 years [1, 2]. The majority of patients with migraine rely on acute medication to control symptoms, where approximately 38% of patients would benefit from the use of preventive therapies and only 3‒13% actually receive them [3]. Among the patients using preventive migraine treatment, e.g., topiramate and onabotulinumtoxinA, patients with CM discontinue preventive treatment primarily due to side effects and lack of efficacy [3, 4], with a sharp drop in persistence in commonly prescribed oral preventive medication at 30 days and over the ensuing 12 months [5]. Given the enduring effects of migraine, understanding the effects of long-term treatment, particularly on patient-reported outcomes (PROs), is important.

A humanized immunoglobulin G1 monoclonal antibody that binds quickly and durably to calcitonin gene-related peptide, which is integral in migraine pathophysiology [6–8], eptinezumab is approved for the preventive treatment of episodic and chronic migraine in adults [9]. The two pivotal phase 3 trials, PROMISE-1 in patients with episodic migraine and PROMISE-2 in patients with CM, determined that intravenous (IV) infusion of 100 mg and 300 mg achieved the primary efficacy endpoint by significantly decreasing mean monthly migraine days over Weeks 1‒12 [10, 11].

The PREVAIL study evaluated the long-term safety, immunogenicity, and impact on PROs of repeated doses of eptinezumab in patients with CM, demonstrated a favorable safety profile, and provided early and sustained reductions in overall migraine-related burden [12]. The favorable safety profile of eptinezumab demonstrated in this long-term trial is consistent with previously published results [13, 14]. The objective of this post hoc analysis was to evaluate item-level changes in the Migraine Disability Assessment (MIDAS) questionnaire over 2 years in patients with CM on eptinezumab treatment. Additional objectives of this analysis included examining correlations between MIDAS total score and migraine days and between MIDAS scores and other PROs (6-item Headache Impact Test [HIT-6], Patient Global Impression of Change [PGIC]) in order to further support the clinical relevance of MIDAS in patients with CM.

## Methods

### Data source

The detailed methodology for PREVAIL has been reported [12]. In brief, PREVAIL was a phase 3, open-label study conducted at 20 study sites in the United States from 12 December 2016 to 11 April 2018 that evaluated the long-term safety, immunogenicity, and impact on PROs of repeated doses of 300 mg IV eptinezumab administered over 30 min in 128 adults [12]. In addition, PREVAIL included 2 treatment phases: the primary treatment phase included 4 infusions of eptinezumab 12 weeks apart (Day 0, and Weeks 12, 24, and 36); the secondary treatment phase included up to 4 additional eptinezumab infusions 12 weeks apart (Weeks 48, 60, 72, and 84). Further, patients were followed for 20 additional weeks until Week 104, for a total study duration of 106 weeks, including the screening period.

Patients were between the ages of 18‒65 and had a diagnosis of migraine at $\le$ 50 years of age with history of CM $\ge$ 1 year (International Classification of Headache Disorders, 3rd edition, beta [ICHD-3β] criteria) [15]. In addition, patients had been prescribed or recommended by a healthcare professional to use prescription or over-the-counter medication for acute and/or prophylactic treatment of migraine, and any prophylactic use of medications for headaches was stable for ≥ 3 months prior to screening. All clinical work was in compliance with current Good Clinical Practices as outlined in the International Conference on Harmonisation of Technical Requirements for Registration of Pharmaceuticals for Human Use, local regulatory requirements, and the principles of the Declaration of Helsinki. All participants provided written informed consent prior to participation.

### Outcomes and assessments

PRO measures included the MIDAS questionnaire [12], which measured the headache effect on patient daily functioning. The MIDAS questionnaire was administered on Day 0, at Week 12, and every 12 weeks thereafter until Week 104. Specifically, MIDAS is composed of five questions that assessed the patient’s performance over the past 3 months (i.e., 12-week recall), where the response to each question was provided in number of days, which were then totaled to determine the level of disability: 0‒5, MIDAS grade I (little or no disability); 6‒10, MIDAS grade II (mild disability); 11‒20, MIDAS grade III (moderate disability); and 21‒40, MIDAS grade IV (severe disability) [16]. Due to the high number of patients included within the severe disability category, this category was further subdivided into a fifth category, 41‒270, MIDAS grade V (very severe disability) [17]. Within the MIDAS questionnaire, items 1, 3, and 5 assess absenteeism, or how many days the patient missed work/school (Question 1), household work (Question 3), or family/social/leisure activities (Question 5), whereas items 2 and 4 measure presenteeism, or how many days the patient had reduced productivity in work/school (Question 2) or household work (Question 4) [18]. Two supplementary items assessed the number of headache days, i.e., MIDAS headache days in the past 3 months (if a headache lasted more than 1 day, each day was counted), and average headache pain severity (from 0 [no pain at all] to 10 [pain as bad as it can be]). A meaningful threshold for change in MIDAS total score is a reduction of $\ge$ 5 points (days) in total score when a baseline score is 11‒20 days and $\ge$ 30% with a baseline score of > 20 days [19].

In addition, the PGIC was administered at Weeks 4, 8, 12, and every 12 weeks thereafter until Week 104. The PGIC includes a single question concerning the patient’s impression of change in their disease status since the start of the study, with seven potential answers: very much improved, much improved, minimally improved, no change, minimally worse, much worse, and very much worse [12]. The HIT-6 was administered at screening, on Day 0, at Weeks 4 and 12, and every 12 weeks thereafter until Week 104 [12]. Specifically, HIT-6 measures the impact of migraine on daily life, comprising six items: severe pain, social limitations, role limitations, cognitive functioning (4-week recall), psychological distress (4-week recall), and vitality (4-week recall) using a Likert scale of frequency: never (6), rarely (8), sometimes (10), very often (11), and always (13). The total scores range from 36‒78, where a 6-point decrease is considered clinically meaningful in patients with chronic migraine [20].

### Statistical analysis

The safety population included all patients receiving $\ge$ 1 dose of eptinezumab [12]. MIDAS, HIT-6, and PGIC scores were summarized using descriptive statistics by timepoint with no imputation for missing values. To examine the relationships among MIDAS, HIT-6, and PGIC scores, Spearman correlations were calculated at Week 12. All analyses were conducted using SAS software (SAS Institute, Cary, North Carolina) Version 9.2 or higher.

## Results

### Study population

All 128 patients enrolled in the study received $\ge$ 1 dose of 300 mg eptinezumab, where 125 patients (97.7%) remained in the study until Week 12, 118 patients (92.2%) attended the Week 48 visit, and 100 patients (78.1%) attended the Week 104 visit. The majority of patients (67.2%) received all 8 doses of eptinezumab and 87.5% received $\ge$ 4 doses [12]. Patients were predominately female (85.2%), white (95.3%), and an ethnicity other than Hispanic or Latino (79.7%); baseline demographic information is reported in Table 1. In the 3 months prior to screening, the mean number of migraine and headache days per 28 days among patients was 14.1 and 20.3, respectively [12]. In addition, at baseline, 38.3% of patients had a diagnosis of medication-overuse headache (ICHD-3β criteria) [12, 15].

**Table 1:** Baseline demographics

	Eptinezumab 300 mg (*N* = 128)
Mean (SD) age, years	41.5 (11.33)
Sex, *n* (%)	
Male	19 (14.8)
Female	109 (85.2)
Ethnicity, *n* (%)	
Hispanic or Latino	26 (20.3)
Not Hispanic or Latino	102 (79.7)
Race, *n* (%)	
White	122 (95.3)
Black or African American	4 (3.1)
Asian	1 (< 1)
Multiple races	1 (< 1)

*SD* Standard deviation

### MIDAS headache days and migraine pain severity

**Fig. 1:**
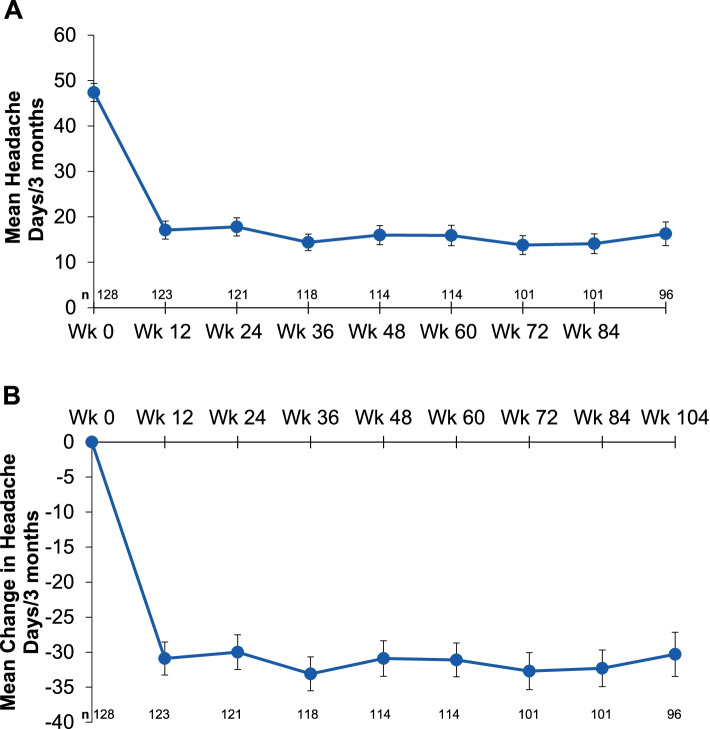
MIDAS headache days* across 2 years: **A** mean headache days ± standard error, and **B** mean change ± standard error in headache days. MIDAS, Migraine Disability Assessment. *Number of headache days occurring over 3-month (12-week) periods over the course of the trial

**Fig. 2:**
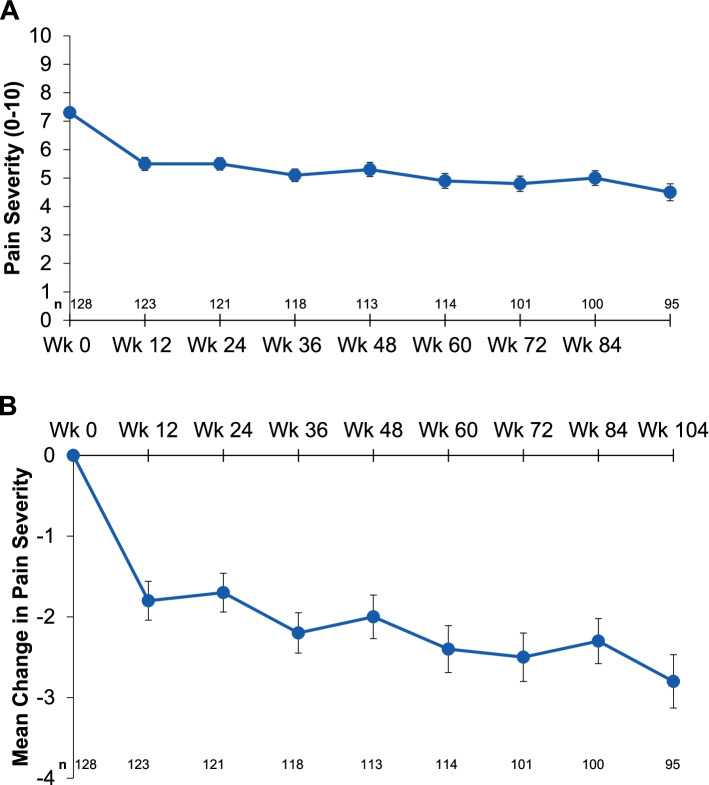
MIDAS pain severity* over 2 years: **A** mean pain severity score ± standard error, and **B** mean change in pain severity score ± standard error. MIDAS, Migraine Disability Assessment. *Pain severity graded on 0‒10 scale

Eptinezumab reduced mean headache days, as assessed by MIDAS, over a 3-month period from 47.4 at baseline to 17.1 at Week 12, which was sustained to Week 104 (mean 16.3 days) (Fig. 1). Further, the average MIDAS headache pain severity (graded on a 0‒10 scale) was reduced from a mean of 7.3 at baseline to 5.5 at Week 12 and to 4.5 at Week 104 (Fig. 2).

### MIDAS item scores over 2 years

**Fig. 3:**
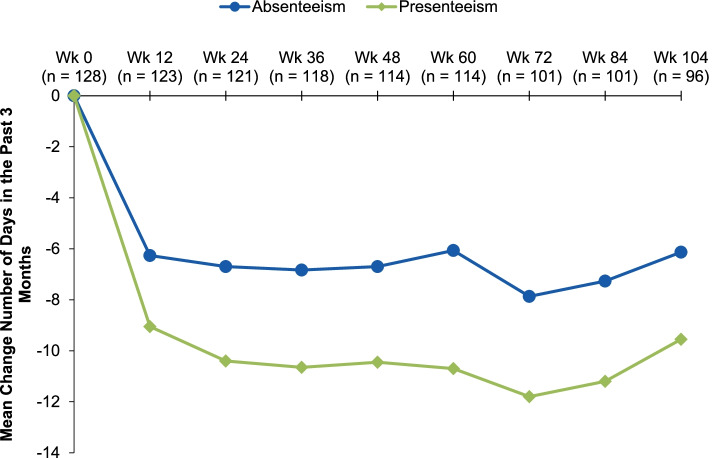
Change from baseline in MIDAS item scores measuring absenteeism* and presenteeism^†^ over 2 years. MIDAS, Migraine Disability Assessment. *Absenteeism comprises the average of Items 1, 3, and 5. Item 1: missed work/school; Item 3: no household work; Item 5: missed family/social/leisure activities. The average at baseline was 9.7 ^†^Presenteeism comprises the average of Items 2 and 4. Item 2: work/school productivity ≤ half; Item 4: household productivity ≤ half. The average at baseline was 14.2

At baseline, the greatest MIDAS disability was noted in Question 4 (mean 16.4 days) and Question 3 (mean 15.6 days), which was reduced to means of 5.5 and 4.7 days, respectively, at Week 12. Mean scores for Questions 1, 2, and 5 were reduced from 5.4, 12.0, and 8.0 days at baseline to 2.2, 4.8, and 2.8 at Week 12, respectively. Reductions at Week 12 were generally sustained through Week 104 (Table 2**, **Supplemental Fig. 1). The mean MIDAS scores measuring absenteeism (Questions 1, 3, 5) changed from 9.7 days at baseline to 3.2 days (Week 12, *n* = 123) to 3.9 days (Week 104, *n* = 95). Mean MIDAS scores measuring presenteeism (Questions 2, 4) decreased from 14.2 days at baseline to 5.2 days at Week 12 (*n* = 123) and was sustained at 5.2 days at Week 104 (*n* = 95) (Fig. 3).

**Table 2:** MIDAS scores* at baseline, Week 12, and Week 104

		**Baseline**(*n* = 128)	**Week 12** (*n* = 123)	**Week 104** (*n* = 95)
Question A: On how many days in the last 3 months did you have a headache? (If a headache lasted more than 1 day, count each day.)	Mean CFB	47.4 –	17.1 ‒30.9	16.3 ‒30.3
Question B: On a scale of 0–10, on average how painful were these headaches? (Where 0 = no pain at all, and 10 = pain as bad as it can be.)	Mean CFB	7.3 –	5.5 ‒1.8	4.5 ‒2.8
Item 1: On how many days in the last 3 months did you miss work or school because of your headaches?	Mean CFB	5.4 –	2.2 ‒2.9	2.4 ‒3.4
Item 2: How many days in the last 3 months was your productivity at work or school reduced by half or more because of your headaches? (Do not include days you counted in question 1 where you missed work or school.)	Mean CFB	12.0 –	4.8 ‒7.1	4.7 ‒7.8
Item 3: On how many days in the last 3 months did you not do household work (such as housework, home repairs and maintenance, shopping, caring for children and relatives) because of your headaches?	Mean CFB	15.6 –	4.7 ‒10.9	5.8 ‒10.2
Item 4: How many days in the last 3 months was your productivity in household work reduced by half or more because of your headaches? (Do not include days you counted in question 3 where you did not do household work.)	Mean CFB	16.4 –	5.5 ‒11.0	5.6 ‒11.3
Item 5: On how many days in the last 3 months did you miss family, social or leisure activities because of your headaches?	Mean CFB	8.0 –	2.8 ‒5.0	3.6 ‒4.8

*CFB* Change from baseline, *MIDAS* Migraine Disability Assessment

### MIDAS total score from baseline to week 104

**Fig. 4:**
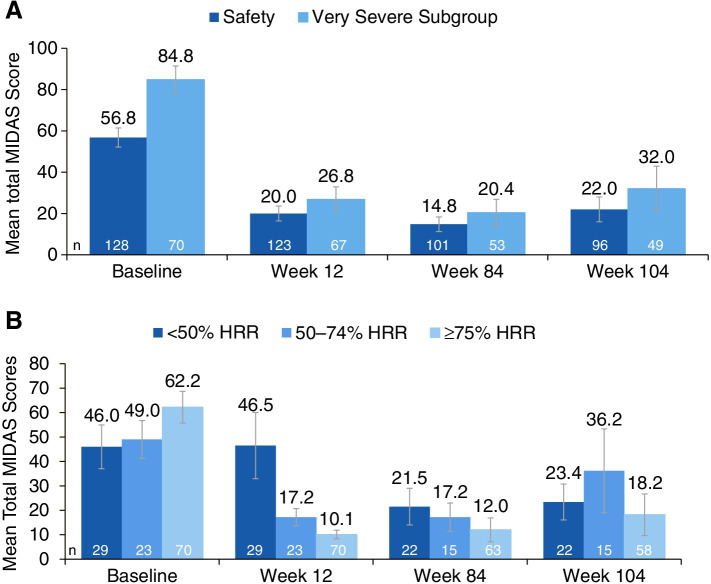
MIDAS total score ± standard error at baseline, Week 12, Week 84, and Week 104: **A** total safety population and subgroup with very severe MIDAS disability*, and **B** patients with < 50%, 50‒74%, or ≥ 75% HRR at Week 12. HRR, headache responder rate assessed by MIDAS; MIDAS, Migraine Disability Assessment. *Level of disability based on MIDAS total score: little or no disability (0–5), mild disability (6–10), moderate disability (11–20), severe disability (21–40), very severe disability (41–270)

Patients who were categorized with very severe MIDAS disability (scores ≥ 41) had a mean total MIDAS score of 84.8, with an average reduction of 56.7 days at Week 12, which was sustained at 32 days at Week 104 (Fig. 4A). At baseline, patients who were < 50%, 50‒74%, and ≥ 75% monthly headache responders had a mean total MIDAS score of 46.0, 49.0, and 62.2 days, respectively, that changed to 46.5, 17.2, and 10.1 by Week 12, and by 23.4, 36.2, and 18.2 by Week 104 (Fig. 4B).

### Correlations among patient-reported outcomes

The percentage of patients reporting “very much improved” or “much improved” PGIC increased from 61.1% at Week 4 to 81.0% at Week 48 and was maintained throughout the remainder of the study [12]. The mean (standard deviation) HIT-6 scores were 65.2 (4.76) at baseline, 57.1 (8.15) at Week 4, 56.9 (8.69) at Week 48, and 56.1 (9.07) at Week 104 [12]. At Week 12, MIDAS headache days were moderately correlated with MIDAS total score (*r *= 0.5937) and PGIC (*r* = 0.4679) and weakly correlated with HIT-6 total score (*r* = 0.3995).

## Discussion

In this post hoc analysis, repeated doses of eptinezumab led to clinically meaningful improvement in total MIDAS score and in MIDAS-measured headache pain severity and mean number of headache days, an effect that was observed at the first post-baseline assessment (at 12 weeks) and sustained after each additional dose. This early efficacy finding is consistent with a previous post hoc analysis of data from PROMISE-2, where more patients treated with eptinezumab than those receiving placebo responded as early as month 1 following infusion [21]. Additionally, eptinezumab has been associated with rapid improvements of health-related quality of life, even when administered during a migraine attack, with eptinezumab significantly improving PROs after 4 weeks compared with placebo [22]. The results reported here support and are consistent with previously published results from the double-blind, placebo-controlled PROMISE-2 trial demonstrating that the preventive treatment effect of eptinezumab significantly reduces monthly migraine days from baseline relative to placebo (100 mg, ‒7.7 days, 300 mg, ‒8.2 days; placebo, ‒5.6 days), is sustained over a full 24 weeks, and has an acceptable safety profile in patients with chronic migraine [11, 23].

In PREVAIL, eptinezumab reduced mean monthly headache frequency as quantified by MIDAS from 15.8 monthly headache days at baseline (47.4 headache days over previous 3 months) to 5.7 monthly headache days at Week 12 (17.1 headache days over previous 3 months), which was sustained to Week 104 (5.4 monthly headache days; 16.3 headache days over previous 3 months). These reductions in monthly headache days throughout the study mirrored the reduction in average MIDAS headache pain severity from baseline to Week 104.

Further, the MIDAS scores assessing absenteeism (Questions 1, 3, and 5) or how many days the patient missed work/school/family activities, decreased ~ 6 days from baseline to Week 104, whereas the scores measuring presenteeism (Questions 2 and 4) or how many days the patient had reduced productivity in work or school, decreased ~ 9 days from baseline to Week 104; the slightly lower efficacy seen at Week 104 is likely due to the time from last eptinezumab dose (20 weeks). Overall, these data suggest that eptinezumab treatment may increase patient productivity and engagement in everyday life. In addition, these reductions in MIDAS total score were most pronounced in patients categorized as “very severe” at baseline, suggesting additional benefits for patients most affected by CM [17].

MIDAS, HIT-6, and PGIC are all PRO measures that are recognized by the International Headache Society as valid instruments to quantify patient satisfaction and headache-related healthcare outcomes [24], and all demonstrated improvements in health-related quality of life (HRQoL) that were sustained throughout the course of the study. In addition, the number of headache days assessed by supplemental questions on MIDAS were moderately correlated to MIDAS total scores and PGIC scores and weakly correlated to HIT-6 scores, providing modest validation of the use of the MIDAS questionnaire to assess headache severity in patients.

Overall, these findings demonstrating sustained reductions in headache pain severity in patients treated with eptinezumab are important due to the high frequency of disability, higher healthcare costs, and reduced HRQoL attributed to migraine overall, particularly in patients with CM [17, 25–29]. Further, given that many patients with CM discontinue preventive migraine treatment [5], this study indicates that long-term treatment with eptinezumab provides sustained reduction of headache frequency and severity and, relatedly, high treatment persistency, with 78.9% of patients receiving eptinezumab treatment through Week 84.

## Limitations

Because this was a post hoc analysis, additional prospectively designed trials are required to confirm these findings. In addition, PREVAIL was not a placebo-controlled study, which limits the interpretation regarding the clinical relevance and internal validation [12]. Further, individuals were excluded from PREVAIL if they had a history or diagnosis of a headache or migraine disorder that did not meet the ICHD-3β criteria for CM, required botulinum toxin injections for any medical/cosmetic reasons within 4 months prior to screening, or had pre-existing significant cardiovascular disease [12], which limits the ability to generalize these results to all adults with CM. Finally, the number of headache days as quantified by MIDAS was based on patient recall rather than real-time recording of headache days using an electronic headache diary.

## Conclusion

Long-term treatment with eptinezumab in patients with CM provided profound and sustained reductions in migraine-related disability as assessed by MIDAS, consistent with the sustained reduction of headache day frequency and pain severity in response to treatment.

## Supplementary Information

**Additional file 1.**

## Data Availability

In accordance with EFPIA’s and PhRMA’s “Principles for Responsible Clinical Trial Data Sharing” guidelines, Lundbeck is committed to responsible sharing of clinical trial data in a manner that is consistent with safeguarding the privacy of patients, respecting the integrity of national regulatory systems, and protecting the intellectual property of the sponsor. The protection of intellectual property ensures continued research and innovation in the pharmaceutical industry. Deidentified data are available to those whose request has been reviewed and approved through an application submitted to https://www.lundbeck.com/global/our-science/clinical-data-sharing.

## Abbreviations

CM: Chronic migraine
ICHD-3β: International Classification of Headache Disorders, 3rd edition, beta
IV: Intravenous
HIT-6: 6-Item Headache Impact Test
HRQoL: Health-related quality of life
MIDAS: Migraine Disability Assessment
PGIC: Patient Global Impression of Change
PRO: Patient-reported outcome
