# Supplementary figures and images for: Long-term reductions in disease impact in patients with chronic migraine following preventive treatment with eptinezumab
Source: BMC Neurol. 2022 Jul 8;22:251. doi: 10.1186/s12883-022-02774-3 (PMC9264513; doi:10.1186/s12883-022-02774-3)

## Slide 1
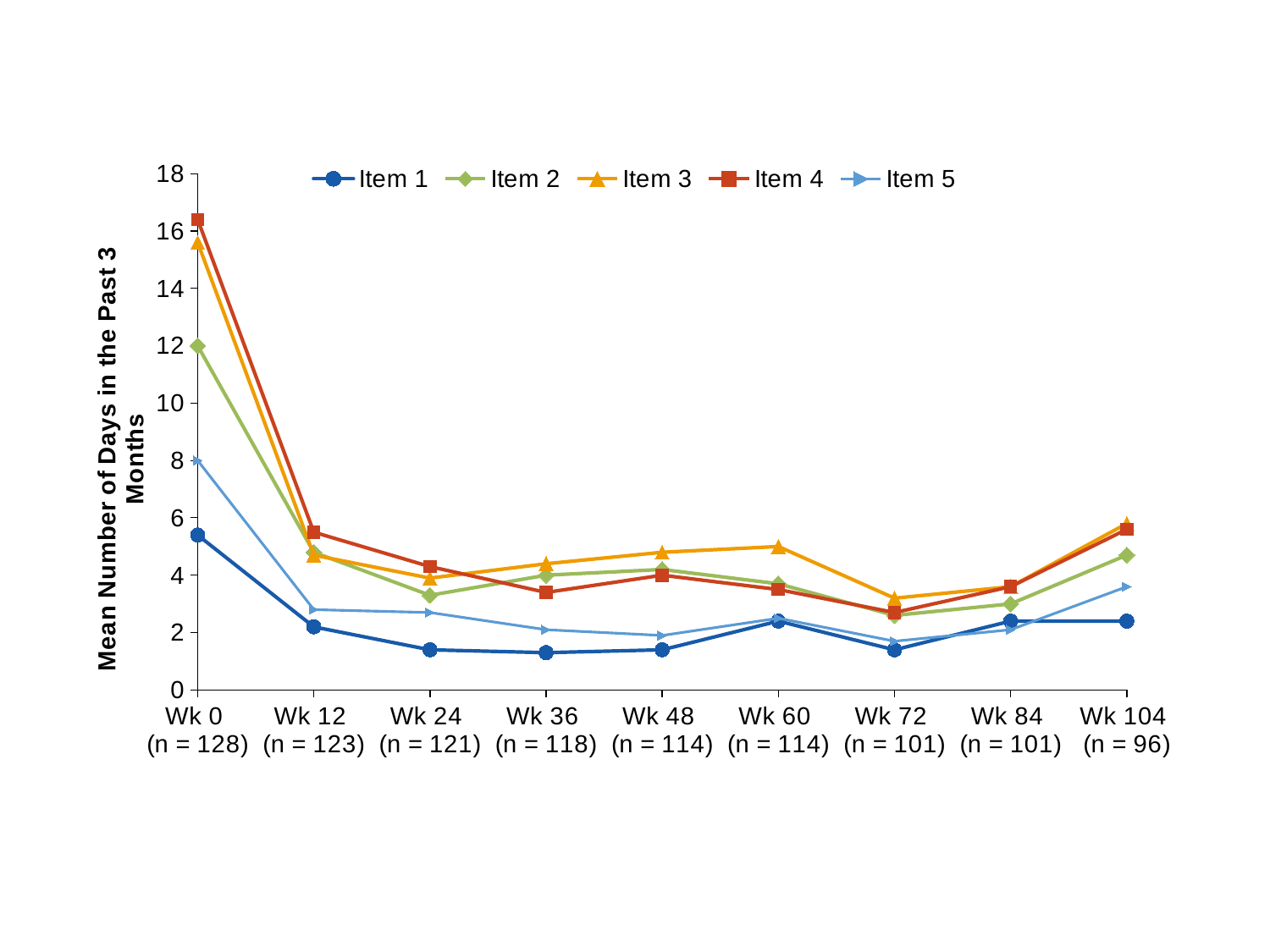

### Chart
| Category | Item 1 | Item 2 | Item 3 | Item 4 | Item 5 |
|---|---|---|---|---|---|
| Wk 0
(n = 128) | 5.4 | 12.0 | 15.6 | 16.4 | 8.0 |
| Wk 12
(n = 123) | 2.2 | 4.8 | 4.7 | 5.5 | 2.8 |
| Wk 24
(n = 121) | 1.4 | 3.3 | 3.9 | 4.3 | 2.7 |
| Wk 36
(n = 118) | 1.3 | 4.0 | 4.4 | 3.4 | 2.1 |
| Wk 48
(n = 114) | 1.4 | 4.2 | 4.8 | 4.0 | 1.9 |
| Wk 60
(n = 114) | 2.4 | 3.7 | 5.0 | 3.5 | 2.5 |
| Wk 72
(n = 101) | 1.4 | 2.6 | 3.2 | 2.7 | 1.7 |
| Wk 84
(n = 101) | 2.4 | 3.0 | 3.6 | 3.6 | 2.1 |
| Wk 104
(n = 96) | 2.4 | 4.7 | 5.8 | 5.6 | 3.6 |

Supplement: Supplementary file 1 — Additional file 1. [file 12883_2022_2774_MOESM1_ESM.pptx]
